# Variations in factors associated with healthcare providers’ intention to engage in interprofessional shared decision making in home care: results of two cross-sectional surveys

**DOI:** 10.1186/s12913-020-5064-3

**Published:** 2020-03-12

**Authors:** Rhéda Adekpedjou, Julie Haesebaert, Dawn Stacey, Nathalie Brière, Adriana Freitas, Louis-Paul Rivest, France Légaré

**Affiliations:** 1 Centre de recherche sur les soins et les services de première ligne de l’Université Laval, Quebec, Canada; 2 Université de Lyon, Université Claude Bernard Lyon 1, Université Saint-Étienne, HESPER EA 7425, F-69008 Lyon, F-42023 Saint-Etienne, France; 3 Ottawa Hospital Research Institute and Faculty of Health Sciences, University of Ottawa, Ottawa, Canada; 4 Centre intégré universitaire en santé et services sociaux de la Capitale-Nationale, Quebec, Canada; 5 Department of Mathematics and Statistics, Université Laval, Quebec, Canada; 6 Canada Research Chair in Shared Decision Making and Knowledge Translation, 2525, chemin de la Canardière, Quebec, G1J 0A4 Canada

**Keywords:** Interprofessional shared decision-making, Home care, Context, Implementation, Clustered randomised trial, Socio-cognitive theory

## Abstract

**Background:**

DOLCE (Improving Decision making On Location of Care with the frail Elderly and their caregivers) was a post-intervention clustered randomised trial (cRT) to assess the effect of training home care teams on interprofessional shared decision-making (IP-SDM). Alongside the cRT, we sought to monitor healthcare providers’ level of behavioural intention to engage in an IP-SDM approach and to identify factors associated with this intention.

**Methods:**

We conducted two cross-sectional surveys in the province of Quebec, Canada, one each at cRT entry and exit. Healthcare providers (e.g. nurses, occupational therapists and social workers) in the 16 participating intervention and control sites self-completed an identical paper-based questionnaire at entry and exit. Informed by the Integrated model for explaining healthcare professionals’ clinical behaviour by Godin et al. (2008), we assessed their behavioural intention to engage in IP-SDM to support older adults and caregivers of older adults with cognitive impairment to make health-related housing decisions. We also assessed psychosocial variables underlying their behavioural intention and collected sociodemographic data. We used descriptive statistics and linear mixed models to account for clustering.

**Results:**

Between 2014 and 2016, 271 healthcare providers participated at study entry and 171 at exit. At entry, median intention level was 6 in a range of 1 (low) to 7 (high) (Interquartile range (IQR): 5–6.5) and factors associated with intention were social influence (β = 0.27, *P* <  0.0001), beliefs about one’s capabilities (β = 0.43, *P* <  0.0001), moral norm (β = 0.31, *P* <  0.0001) and beliefs about consequences (β = 0.21, *P* <  0.0001). At exit, median intention level was 5.5 (IQR: 4.5–6.5). Factors associated with intention were the same but did not include moral norm. However, at exit new factors were kept in the model: working in rehabilitation (β = − 0.39, *P* = 0.018) and working as a technician (β = − 0.41, *P* = 0.069) (compared to as a social worker).

**Conclusion:**

Intention levels were high but decreased from entry to exit. Factors associated with intention also changed from study entry to study exit. These findings may be explained by the major restructuring of the health and social care system that took place during the 2 years of the study, leading to rapid staff turnover and organisational disturbance in home care teams. Future research should give more attention to contextual factors and design implementation interventions to withstand the disruption of system- and organisational-level disturbances.

**Trial registration:**

Clinicaltrials.gov (NCT02244359). Registered on September 19th, 2014.

## Background

When older adults lose autonomy and need more care, they face a decision about staying at home or moving into a nursing home. Although older adults with loss of autonomy should be the principal decision-makers about this choice, cognitive impairment may result in their caregivers making the decision instead [1]. Informal caregivers (eg. family members) play an essential role in caring for cognitively-impaired older adults [2]. However, studies show that they need more support in decision-making and more opportunity to participate in housing decisions made for their cognitively-impaired loved ones [3]. While caregivers are often the experts on the older adult’s condition, history and care experiences [4, 5], they report negative experiences regarding this decision-making process, the choice, and the decision outcomes, possibly because of a lack of effective decision support [1, 6].

Home care enables a person to stay at home or return home quickly after an episode of care [7]. In 2015/2016, an estimated 3.3% of Canadians aged 18 or older (919,000 people) had received home care services in the past year, of whom 511,500 were over 65 years old [8]. For interprofessional home care teams caring for older adults with loss of autonomy, interprofessional shared decision-making (IP-SDM) is a promising way to approach decision-making about housing [9, 10]. The IP-SDM model combines shared decision-making (SDM) [11] with interprofessional collaboration [12, 13]. From 2014 to 2016, we undertook a clustered randomised trial (cRT) of an IP-SDM training program called the DOLCE study (Improving Decision-making On Location of Care with the frail Elderly and their caregivers). DOLCE assessed the effect of giving the training in 16 home care teams in the Province of Quebec on informal caregivers’ participation in the health-related housing decisions of the older adults they cared for [14].

According to the results of the cRT [15], training the home care team in IP-SDM did seem to increase caregivers’ participation in their loved ones’ housing decisions [15]. We had hypothesised that this outcome would occur because post-training, the healthcare providers would involve caregivers more in the decision-making and give them more support. As we were also interested in the mechanisms behind this expected behaviour change in the healthcare providers, we used an integrated model of socio-cognitive variables to monitor the level of behavioural intention to engage in an IP-SDM approach at cRT entry and exit and to identify factors associated with this intention [16].

### Theoretical background

The medical community is increasingly aware of the SDM approach and SDM is now part of health policies in many countries [17]. Yet healthcare providers are slow to adopt it [18]. Behaviour change interventions are essential for improving the practice of clinical medicine, and a thorough understanding of the mechanisms underlying behaviour change is necessary for developing and improving SDM interventions [19, 20]. Socio-cognitive theories provide validated constructs and measures for exploring the mechanisms that drive behaviour change [21].

**Fig. 1:**
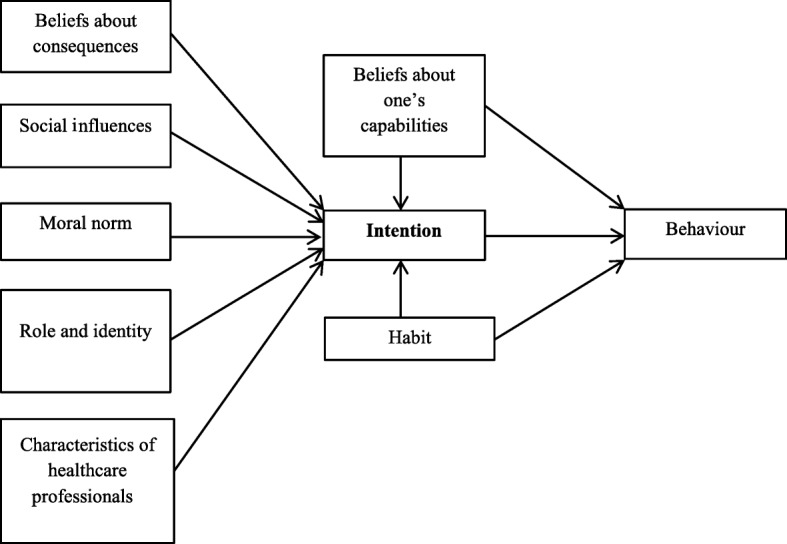
Integrated model for explaining healthcare professionals’ clinical behaviour

Authors of a systematic review of socio-cognitive theories for studying healthcare providers’ clinical behaviour [16] proposed an integrated model that combined the variables they found most important (Fig. 1). We chose this model because it was derived from an extensive systematic review of socio-cognitive theories that included 76 studies and also because it integrates validated socio-cognitive theories with the highest overall efficacy for predicting intention such as the Theory of Planned Behaviour (59% of the variance of intention was explained by the model) and the Triandis’ Theory of Interpersonal Behaviour (81% of the variance of intention was explained by the model). According to this model, the three most important psychosocial factors for predicting behaviour are habit or past behaviour, intention, and beliefs about one’s capabilities (perceptions of facilitators and barriers to adopting the behaviour), with the latter two being most significant. The model also identifies the five most significant predictors of intention as beliefs about one’s capabilities, beliefs about consequences (usefulness and benefits/risks of adopting the behaviour), moral norm (feeling of personal obligation to adopt the behaviour), social influences (perception of approval or disapproval by significant persons regarding adopting the behaviour), and role and identity (beliefs about whether the behaviour should be adopted by someone of a similar age, sex or social position as oneself).

We therefore measured healthcare providers’ intention (and its theory-based predictors) to engage in an IP-SDM approach. This study adheres to STROBE guidelines for reporting cross-sectional studies [22].

## Methods

### Parent study design

Between April 2014 and August 2016, we conducted a two-arm cluster randomised trial with interprofessional home care teams in 16 health and social services centres (HSSCs) in seven health jurisdictions in the province of Quebec, Canada. Details of the study protocol can be found elsewhere [14]. Briefly, study participants were the HSSCs, their interprofessional home care teams, and caregivers of their elderly clients with loss of autonomy and cognitive impairment. The intervention consisted of training in the IP-SDM approach (an online tutorial and a live interactive workshop) and in use of a decision guide. The primary outcome was caregivers’ self-reported role in decision-making. At the entry into the study and after obtaining informed consent, a self-administered questionnaire was given to each member of the interprofessional home care team of the 16 HSSCs to measure their intention (and its determinants) to adopt an SDM approach. Healthcare providers in the HSSCs allocated to the intervention group participated in the training but not those in the control group. The same questionnaire was given again to all participants (both the intervention and control groups) at study exit. This paper reports on data collected during these two cross-sectional surveys at study entry and exit. As the parent trial showed no difference in intention pre-post intervention between the control group and the intervention group (and this trial was neither designed nor powered for that), we chose to consider the data as two separate cross-sectional surveys (alongside the trial), rather than as pre-post intervention data. We took this approach to capture how external factors may have affected the intention to engage in IP-SDM (and its theory-based predictors) over time.

### Setting and participants

Eligible interprofessional home care teams a) were involved in caring for older adults with loss of autonomy, and b) practised in one of the participating HSSCs. A minimum of two healthcare providers from different professions had to be involved in the older adult’s care (definition of an interprofessional approach). Only one interprofessional home care team per HSSC was invited to participate. The selection of the teams was done by home care managers. The way teams were selected varied from one setting to another. The teams were chosen for different reasons: the stability of the team, the number of clients served per week (to be able to recruit the required number of clients in the trial), the budget allocated for the team to participate in the study (for the time spent in the IP-SDM training and the time spent in recruiting clients), and the fact that the team had not participated in the pilot version of the study. There was no financial compensation for the participants.

### Data collection and variables

Data collection took place before the trial in February 2015 and after the trial in October 2016. Data was collected anonymously from healthcare providers using a paper-based self-reported questionnaire: the CPD-Reaction Questionnaire [23]. This validated instrument [23, 24] followed a strict development procedure [23]. It assesses the impact of training on clinical behavioural intention using items based on our socio-cognitive model [16]. The first section defines the targeted behaviour, i.e. engaging in IP-SDM with elderly home care clients and their caregivers who will be facing a health-related housing decision in the next 6 months. The second assesses five constructs: intention (two items), beliefs about one’s capabilities (three items), beliefs about consequences (two items), social influence (three items) and moral norm (two items). Scores per construct range from 1 (low) to 7 (high) (see Additional file 1 for more details). “Habit” and “role and identity” were missing from the variables because they are not assessed by the CPD-Reaction Questionnaire. During its development, items concerning “habit” were removed because they were poorly worded and did not reflect our integrated model’s definition of “habit”. Items concerning “role and identity” were also removed because none of them loaded on any factors as defined by exploratory factor analysis [23]. The Cronbach’s alpha coefficient in entry and exit questionnaires was 0.77 and 0.86 respectively for intention, 0.73 and 0.77 for social influence, 0.79 and 0.86 for beliefs about capabilities, 0.74 and 0.61 for moral norm, and 0.83 and 0.86 for beliefs about consequences. The third section collects sociodemographic characteristics: date of birth, sex, mean number of clients served per week and profession. Possible professions were social worker, nurse, occupational therapist, physiotherapist, physician and other (specified). The dependent variable was the healthcare professionals’ intention to engage in IP-SDM. Our independent variables were beliefs about one’s capabilities, beliefs about consequences, social influence, moral norm and sociodemographic characteristics.

### Statistical analysis

We used descriptive statistics to describe the level of the intention to engage in IP-SDM and the four other psychosocial constructs at study entry and study exit and to describe sociodemographic characteristics of healthcare providers. For profession, the response category “other” contained several types of profession that were not classifiable in the five other response categories. We therefore created a new variable using the National Occupational Classification (NOC) of Canada [25] to achieve more homogenous professional groups: 1) nurses, 2) rehabilitation team (e.g. physiotherapist, occupational therapist), 3) technicians (e.g. licensed practical nurse, respiratory therapist), 4) social workers, 5) social, community and education paraprofessionals (e.g. community worker, special education technician), and 6) activities coordinators.

For most of the pre-trial and post-trial variables, the proportion of missing data ranged from 0.58% to 1.11%. For the variable “number of clients per week”, the proportion of missing data was 6.64% pre-trial and 5.26% post-trial. We compared the characteristics of the participants who provided data for this variable with the characteristics of those who did not and they appeared to be similar. Missing values for that variable seemed to be missing completely at random (MCAR). For these reasons and given the low level of missing data for all variables, we considered that pairwise deletion would be appropriate to address missing data.

To take the non-independence of the data into account (clustering effect), we used multilevel modelling (linear mixed model). This was done by specifying a random effect at the HSSC level. To identify factors associated with healthcare providers’ intention to engage in IP-SDM at study entry, we first performed bivariate analyses to examine the relationship between the theory-based factors and the intention score (at 0.20 alpha level) [26]. Following bivariate analyses, we performed multilevel multivariate regression analysis using backward elimination for model selection. We used the same approach to identify factors associated with healthcare providers’ intention to engage in IP-SDM at study exit. We computed a study entry model and a study exit model. In the study entry model, intention at entry was regressed on variables measured before the trial. In the study exit model, intention at exit was regressed on variables measured after the trial and on the variable representing the study groups (intervention vs. control). For profession, activities coordinators were not considered in the regression analyses given the weak number of participants (*n* = 2). Due to the exploratory nature of the analysis, a *p*-value of < 0.10 was used as the threshold for statistical significance in the final models [27, 28].

We conducted model diagnostics by assessing multicollinearity, distribution of scaled residuals, homoscedasticity, and influential observations.

We performed the analysis using SAS version 9.4 (SAS Institute Inc., Cary, NC, USA) with the MIXED procedure.

### Ethical issues and parent study registration

Ethics committee review approval was obtained from the Centre Hospitalier Universitaire (CHU) de Québec Multicentre Ethics Committee (approval number MP-CHU-QC-14-001). All participants gave written informed consent. The parent study is registered at clinicaltrials.gov (registration number: NCT02244359).

## Results

### Flow of the trial and characteristics of participants

**Fig. 2:**
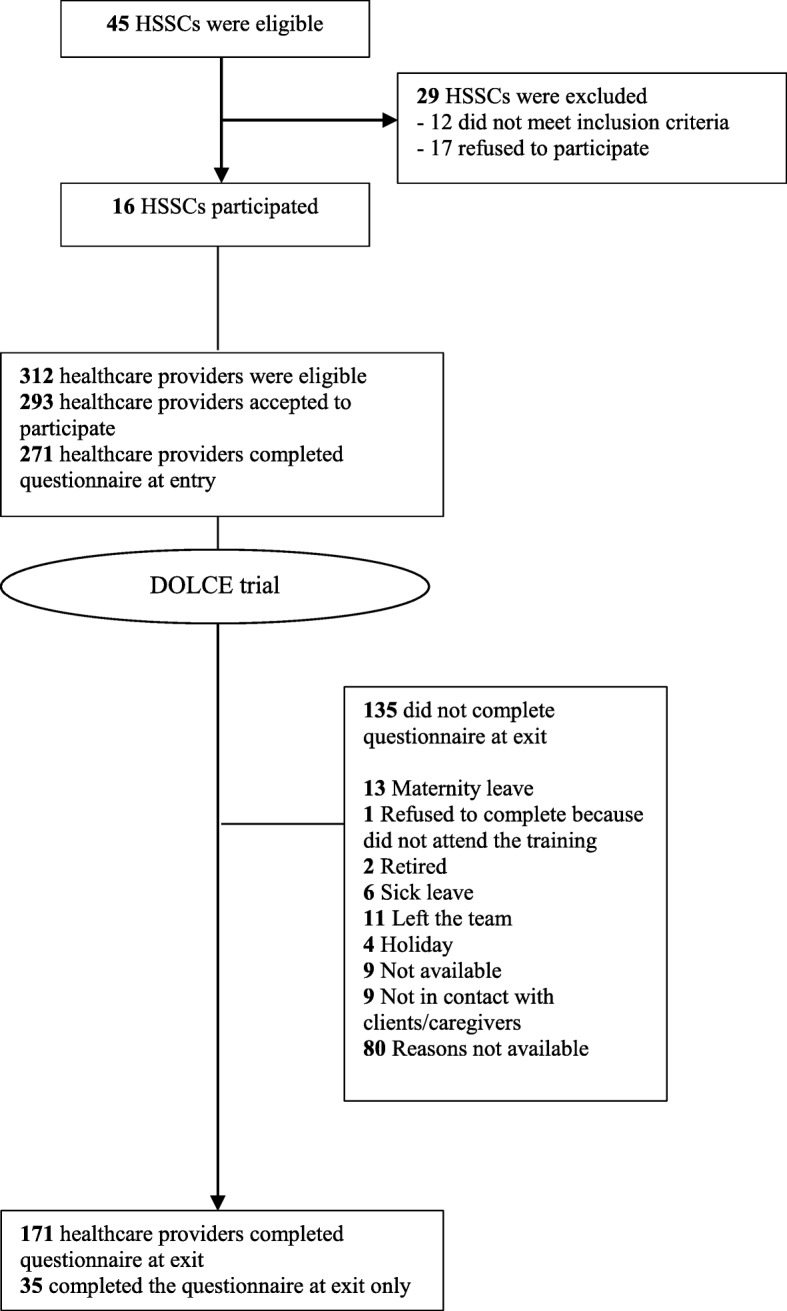
Flow chart of the study

Sixteen HSSCs participated in the study. Totals of 271 and 171 healthcare providers completed the questionnaire at entry and exit respectively (Fig. 2). Participants’ sociodemographic characteristics are reported in Table 1. In both samples, most of the healthcare providers were female (90.4% at entry and 88.9% at exit) with a median age of 36.1 years (Interquartile range (IQR): 30.1–45.9) in the entry sample and 38.6 years (IQR: 31.9–48.1) in the exit sample. Additional file 2 provides scores of the five determinants according to profession. Technicians and activities coordinators reported the lowest levels of intention at study entry and these scores decreased at study exit.

**Table 1:** Characteristics of the participants at study entry and study exit (both intervention and control groups)

Characteristics	At study entry (n 271)	At study exit (*n* = 171)
	Number (percentage)	
**Sex**		
Male	26 (9.6)	18 (10.5)
Female	245 (90.4)	152 (88.9)
Missing	0 (0)	1 (0.6)
**Profession**		
Nurses	61 (22.5)	29 (16.9)
Rehabilitation team	50 (18.5)	34 (19.9)
Technicians	10 (3.7)	7 (4.1)
Social workers	100 (36.9)	75 (43.8)
Social, community & education paraprofessionals	48 (17.7)	23 (13.5)
Activities coordinators	2 (0.7)	2 (1.2)
Missing	0 (0)	1 (0.6)
	Median (interquartile range)	
**Age** (in years)	36.1 (30.1–45.9)	38.6 (31.9–48.1)
**Number of clients served per week**	15 (10–25)	15 (10–20)
**Intention**	6 (5–6.5)	5.5 (4.5–6.5)
**Social influence**	5.5 (5.1–6.2)	5.5 (4.7–6.2)
**Beliefs about capabilities**	5.7 (5–6.3)	5.7 (5–6.3)
**Moral norm**	6.3 (5.5–7)	6.2 (5.5–7)
**Beliefs about consequences**	6 (5.5–7)	6 (5–6.5)

### Healthcare providers’ intention to engage in IP-SDM at study entry and factors associated with intention

Healthcare providers’ scores of intention to engage in IP-SDM at entry was 6 in a range of 1 (low) – 7 (high) (IQR: 5–6.5). In multilevel multivariate regression analyses (Table 2), the factors associated with higher healthcare providers’ intention to engage in IP-SDM were perception of approval by colleagues or significant others in the profession (“social influence”) (β = 0.27, *P* <  0.0001), perceptions of facilitators and barriers to adopting the behavior (“beliefs about their capabilities”) (β = 0.43, *P* <  0.0001), feeling of personal obligation to engage in IP-SDM (“moral norm”) (β = 0.31, *P* <  0.0001) and beliefs about the usefulness and the benefits of engaging in it (“beliefs about consequences”) (β = 0.22, *P* <  0.0001). These factors explained 68.4% of the variance of the intention in the model (*R*^2^).

**Table 2:** Factors associated with healthcare professionals’ intention to use the IP-SDM at study entry (*n* = 269)^a^

Variables	Bivariate analyses^b^			Final model^b^		
	β	95% CI	*p*-value^a^	β	95% CI	*p*-value^╫^
**Age** (in years)	− 0.014	− 0.030 to 0.000	**0.060**	–	–	–
**Sex**						
Female (vs male)	0.87	0.31 to 1.42	**0.004**			
**Number of clients served per week**	−0.014	− 0.026 to − 0.001	**0.027**	–	–	–
**Profession**			0.223			
Nurses (vs Social workers)	− 0.29	− 0.72 to 0.14	0.183	–	–	–
Rehabilitation team (vs Social workers)	−0.17	− 0.63 to 0.28	0.451	–	–	–
Technicians (vs Social workers)	−1.10	−1.94 to −0.24	0.013	–	–	–
Social, community & education paraprofessionals (vs Social workers)	−0.18	−0.66 to 0.29	0.445	–	–	–
**Social influence**	0.87	0.75 to 0.98	**< 0.0001**	0.27	0.14 to 0.39	**< 0.0001**
**Beliefs about capabilities**	0.93	0.83 to 1.03	**< 0.0001**	0.43	0.31 to 0.56	**< 0.0001**
**Moral norm**	0.94	0.84 to 1.05	**< 0.0001**	0.31	0.17 to 0.45	**< 0.0001**
**Beliefs about consequences**	0.82	0.71 to 0.93	**< 0.0001**	0.21	0.10 to 0.33	**< 0.001**
*R*^2^				**68.4%**		

^a^Activities coordinators were not considered in the analyses given the small number of participants (*n* = 2)

^b^Linear mixed regression model with adjustment for clustering

^a^At 0.20 alpha level

^╫^ A *p*-value of < 0.10 was used as the threshold for statistical significance in the final models

“-” This variable was not kept in the final model

### Healthcare providers’ intention to engage in IP-SDM at study exit and factors associated with intention

Healthcare providers’ score of intention to engage in IP-SDM at exit was 5.5 (IQR: 4.5–6.5). In multilevel multivariate regression analyses (Table 3), the same factors were associated with higher intention to engage in IP-SDM except for moral norm (feeling of personal obligation to adopt the behaviour). However, unlike at study entry, new factors were kept in the final model: working in a rehabilitation team (β = − 0.39, *P* = 0.018) or as a technician (β = − 0.41, *p* = 0.069). The associated factors explained 77.1% of the variance of the intention in the model (*R*^2^).

**Table 3:** Factors associated with healthcare professionals’ intention to engage in IP-SDM at study exit (*n* = 169)^a^

Variables	Bivariate analyses^b^			Final model^b^		
	β	95% CI	*p*-value^a^	β	95% CI	*p*-value^╫^
**Age** (in years)	− 0.001	− 0.025 to 0.022	0.929	–	–	–
**Sex**						
Female (vs male)	0.21	−0.65 to 1.08	0.597	–	–	–
**Number of clients served per week**	−0.022	−0.040 to − 0.004	**0.015**	–	–	–
**Profession**			**0.036**^d^			**0.001**
Nurses (vs Social workers)	−0.00	− 0.68 to 0.66	0.982	0.02	−0.25 to 0.29	0.888
Rehabilitation team (vs Social workers)	−0.36	−1.00 to 0.28	0.257	−0.39	−0.72 to − 0.07	**0.018**
Technicians (vs Social workers)	−1.78	−2.99 to − 0.58	0.005	− 0.41	−0.86 to 0.03	**0.069**
Social, community & education paraprofessionals (vs Social workers)	0.21	−0.51 to 0.95	0.547	−0.06	−0.48 to 0.35	0.760
**Social influence**	0.99	0.86 to 1.11	**< 0.0001**	0.34	0.22 to 0.46	**< 0.0001**
**Beliefs about capabilities**	0.99	0.88 to 1.09	**< 0.0001**	0.46	0.25 to 0.68	**< 0.0001**
**Moral norm**	0.87	0.68 to 1.07	**< 0.0001**	–	–	**–**
**Beliefs about consequences**	1.08	0.95 to 1.21	**< 0.0001**	0.42	0.21 to 0.63	**< 0.001**
**Intervention**						
Intervention (vs no intervention)	−0.30	−0.98 to 0.38	0.355	–	–	**–**
*R*^2^				**77.1%**		

^a^Activities coordinators were not considered in the analyses given the small number of participants (*n* = 2)

^b^Linear mixed regression model with adjustment for clustering

^a^At 0.20 alpha level

^d^Overall *p*-value

“-” This variable was not kept in the final model

^**╫**^ A *p*-value of < 0.10 was used as the threshold for statistical significance in the final models

## Discussion

Alongside a clustered randomised trial of an IP-SDM intervention, we monitored healthcare providers’ level of intention to engage in IP-SDM in home care and identified factors associated with intention. Intention levels were high but decreased from entry to exit. At trial entry, we observed that greater social influence, beliefs about capabilities, moral norm, and beliefs about consequences were associated with greater intention to engage in IP-SDM. At exit, the same factors were observed with the exception of moral norm. In addition, we observed that working in rehabilitation or as a technician (compared to working as a social worker) were associated with lower intention. This led us to make the following observations.

First, regarding factors associated with intention, our results are supported by a large body of accumulated evidence. A systematic review of healthcare providers’ intention to use research and products of research in clinical practice reported that attitude, subjective norm and perceived behavioural control (three predictors of intention according to the Theory of Planned Behaviour [29]) were dominant predictors of intention in a range of behaviours [30]. These predictors correspond to beliefs about consequences, social influence and beliefs about capabilities in our integrated model. Another systematic review reported that the same three theory-based variables were most often identified as determinants of healthcare providers’ intention to engage in SDM-related behaviours [31]. Our results suggest that moral norm should be added as another important factor associated with intention to engage in IP-SDM, a result supported by literature relating to a number of different behaviours such as to use a decision aid for Down Syndrome screening in the context of prenatal consultation with a pregnant woman and her partner, to engage preschoolers in physical activity in the context of childcare, and to practice euthanasia for end-of-life patients in the context of palliative care [16, 32–39], especially concerning behaviours with an ethical dimension [35]. This is coherent with increasing recognition that SDM should be considered not just as a pragmatic approach but as an ethical imperative [40]. As stated by Elwyn and colleagues, “the imperative for shared decision-making rests on the principles of good clinical practice, respecting patients’ right to know that their informed preferences should be the basis for healthcare providers’ actions” [41]. In a study of the psychosocial determinants of physicians’ intention to practice euthanasia in palliative care, researchers measured three ethical principles underlying moral norm: autonomy (e.g. believing patients should control when they die), beneficence (e.g. believing euthanasia will provide them relief) and justice (e.g. believing that euthanasia frees resources for others in need) [37]. They found that moral norm in the case of the physicians was related to beneficence. In the case of SDM, it would be interesting to study how autonomy (e.g. believing patients should have more control over their own healthcare decisions), beneficence (e.g. believing that involving patients in decisions about their health care is associated with less decision regret afterwards) or justice (e.g. believing that informed patients will choose less costly options and free up resources for others), are related to moral norm among healthcare providers considering whether to engage in IP-SDM. This knowledge could inform the design of interventions designed to target moral norm.

Second, at study exit, intention scores decreased, moral norm was no longer associated with intention, and additional factors appeared. As at study entry, intention still increased when social influence, beliefs about one’s capabilities and beliefs about consequences increased. But working in the rehabilitation team or as a technician tended to lower intention compared to being a social worker. We also observed that the intention of activities coordinators decreased at exit. As shown by the results, the intervention had no effect on intention. Moreover, the evaluation of the workshop showed that the satisfaction of the healthcare providers who followed the training was high (results not shown). Therefore, the decrease in the level of intention cannot be explained by the implementation of the intervention. This variation in level of intention among team members and in the factors associated with their intention may be explained by staff workload, staff turnover and discouragement caused by major changes in the health and social care system that occurred during the course of the study (2014 – 2015), when the Province of Quebec entirely restructured its health and social care system [42], requiring many providers to take on extra clients. Indeed, as a result of the restructuring, 1394 workers lost their jobs and home care providers experienced a 36.9% increase in patient interventions (2014–2017) [43]. According to our data, rehabilitation workers and technicians were the professional groups that reported an increase in the median number of clients served per week from study entry to study exit (results not shown). Staff workload has been identified as barrier to engaging in IP-SDM [44] and the related lack of time is the most widely reported barrier, as indeed for uptake of many innovations [45]. These upheavals also resulted in high staff turnover, which deeply affected the home care teams who participated in the study. Several participants left the teams, did not complete the questionnaire at exit, and gave no reasons. High staff turnover is frequently identified by healthcare providers as a barrier to engaging in IP-SDM [44]. This factor affects team cohesion and communication and is likely to directly impact the quality of the provider–patient relationship, as well as the relationships among providers [46]. All these organisational disturbances may explain the observed activities coordinators’ lower intention to engage in IP-SDM at exit.

Third, the discouragement experienced by staff as a result of these upheavals may also explain why moral norm did not appear to be a factor associated with intention at exit. Even if healthcare providers still found it acceptable and ethical to engage in IP-SDM, their intention was no longer driven by what they considered morally acceptable or desirable but by practical issues that may have hindered successful interprofessional collaboration. A systematic review of reviews has shown that systemic upheavals can undermine commonly held values or beliefs in a society at a given time, such as evidence-based practice and patient-centred care. Authors highlight the importance of aligning interventions with system characteristics (e.g. policy and legislation, local and national agenda), organizational characteristics (e.g. leadership, organisational readiness, existing workflow, clarity of roles and responsibilities, and division of labour) as well as professional characteristics (e.g. attitudes to change, perception of time and workload) [47]. Indeed most SDM research has focused more on strategies that address professional-level barriers [48] and less on identifying and assessing strategies that address barriers at the organisational and system levels [49]. Organisational contexts needs to be seen as an integral part of behaviour change instead of a source of confounding variables [50] in order to explore how implementation interventions can be designed to withstand the disruptions of system level and organisational level disturbances.

This study has a number of limitations. First, it was embedded in a larger study and was neither designed nor powered for our stated objectives. Second, there were few respondents in some of the provider categories (e.g. technicians and activities coordinators). A bigger sample size would have given us more confidence in the interpretation of our results regarding these groups. Third, it is possible that a social desirability bias occurred because of the increasing popularity of and public pressure for patient involvement in health-related decisions [17], as suggested by the overall high scores of intention and its determinants. Fourth, we collected data at the individual level only. Gathering data at other levels of context (system and organisation) would have had given us deeper insight into how the health and social care reforms influenced the healthcare providers’ level of intention and its associated factors. Fifth, we did not have observational data to assess how these levels of intention to engage in IP-SDM translate into healthcare providers’ adoption of that behaviour. Another limitation is related to the exploratory nature of the analysis. Our analytical approach may have overestimated the measures of association and may have produced false positive tests. However, this approach was not only based on the data, since variables collected were informed by validated socio-cognitive theories. In addition, even if little is known about factors associated with healthcare providers’ intention to engage in IP-SDM, our results are consistent with other evidence on determinants of healthcare providers’ intention to engage in health-related behaviours [16, 29–31]. Finally, more than 100 of the participants who completed the questionnaire at entry did not complete the questionnaire at exit. Completion of the questionnaire at exit by these participants would have given us a larger sample size and a more complete picture of the level of intention at exit and its associated factors. Despite this, the possibility of non-response bias is very low for the following reasons: most non-respondents did not complete the questionnaire at exit because they were on leave, had left the team or lost their jobs, i.e. their non-response was unrelated to their intention to engage in IP-SDM. Second, removing those who did not complete the questionnaire at exit from the entry dataset does not change the results of the analysis, i.e. those who completed the questionnaire at both entry and exit did not differ in meaningful ways from those who did not complete it at exit.

### Implications for research

These findings have several implications for future research. First, as SDM is increasingly recognized as an ethical imperative (and not just empirical), further research is necesssary on the principles underlying moral norm in Theory of Planned Behaviour-related behaviour change studies. Second, as organisation-level changes and related disruptions are regular occurrences in health and social care systems, future implementation interventions need to collect data about the context in which complex interventions are nested and monitor them over time. Analysis of intervention data should include analysis of cross-level linkages between each of the context levels, and between the context and the key evaluation variables [51]. Third, in the design phase of SDM interventions, researchers should plan a prospective process evaluation of the trial to identify barriers and facilitators to implementation that could appear if system- or organisational-level changes occur. Finally, in addition to strategies that address individual-level barriers, research should also focus on identifying and assessing strategies that address organisational and system level barriers.

## Conclusion

From study entry to study exit, the level of healthcare providers’ intention to engage in interprofessonal SDM decreased. Moreover, our study results suggested that factors associated with intention changed from study entry to study exit. This finding may be explained by the major restructuring of the health and social care system that took place during the course of the study. Researchers should give more attention to contextual factors and design implementation interventions that can withstand the disruptions of system-level and organisational-level disturbances.

## Supplementary information

**Additional file 1.** Constructs of the CPD-Reaction questionnaire. This table presents the five psychosocial constructs of the CPD-Reaction questionnaire with their corresponding items and response choices.

**Additional file 2.** Intention to engage in IP-SDM among healthcare professionals by discipline at study entry and exit. This table presents median scores of intention by healthcare professionals’ discipline at study entry and study exit.

**Additional file 3.** STROBE checklist for cross-sectional studies. This table presents a checklist of items that should be included in reports of cross-sectional studies and how this paper adheres to that checklist.

## Data Availability

The datasets used and analysed during the current study are available from the corresponding author on reasonable request.

## Abbreviations

CHU: Centre Hospitalier Universitaire
cRT: Clustered randomised trial
DOLCE: Improving Decision making On Location of Care with the frail Elderly and their caregivers
HSSC: Health and social services centre
IP-SDM: Interprofessional shared decision-making
NOC: National Occupational Classification
SDM: Shared decision-making
