# Supplementary material for: Variations in factors associated with healthcare providers’ intention to engage in interprofessional shared decision making in home care: results of two cross-sectional surveys
Source: BMC Health Serv Res. 2020 Mar 12;20:203. doi: 10.1186/s12913-020-5064-3 (PMC7069220; doi:10.1186/s12913-020-5064-3)
Supplement: Supplementary file 1 — Additional file 1. Constructs of the CPD-Reaction questionnaire. This table presents the five psychosocial constructs of the CPD-Reaction questionnaire with their corresponding items and response choices. [file 12913_2020_5064_MOESM1_ESM.docx]

**Constructs of the CPD-Reaction questionnaire**

| **Construct scale ^a^** | **Item** | **Response choice** |
| --- | --- | --- |
| Intention | I intend to engage in IP-SDM… in the next 6 months | Strongly disagree=1 to strongly agree=7 |
|  | I plan to engage in IP-SDM… in the next 6 months | Strongly disagree=1 to strongly agree=7 |
| Social influence | To the best of my knowledge, the percentage of my colleagues who would engage in IP-SDM… in the next 6 months is | 0-20%  21-40%  41-60%  61-80%  81-100% |
|  | Now think about a colleague a co-worker whom you respect as a professional. In your opinion, would he/she engage in IP-SDM… in the next 6 months? | Never=1 to always=7 |
|  | Most people who are important to me in my profession would engage in IP-SDM… in the next 6 months | Strongly disagree=1 to strongly agree=7 |
| Beliefs about capabilities | I am confident that I could engage in IP-SDM… in the next 6 months if I wanted to | Strongly disagree=1 to strongly agree=7 |
|  | For me, engaging in IP-SDM… in the next 6 months would be | Extremely difficult=1 to easy=7 |
|  | I have the ability to engage in IP-SDM… in the next 6 months | Strongly disagree=1 to strongly agree=7 |
| Moral norm | Engage in IP-SDM… in the next 6 months is the ethical thing to do | Strongly disagree=1 to strongly agree=7 |
|  | It is acceptable to engage in IP-SDM… in the next 6 months | Strongly disagree=1 to strongly agree=7 |
| Beliefs about consequences | Overall, I think that for me engaging in IP-SDM… in the next 6 months would be | Useless=1 to useful=7 |
|  | Overall, I think that for me engaging in IP-SDM… in the next 6 months would be | Harmful=1 to beneficial=7 |

^a^ The score by construct is obtained by computing the mean score of the items
