# Supplementary material for: Variations in factors associated with healthcare providers’ intention to engage in interprofessional shared decision making in home care: results of two cross-sectional surveys
Source: BMC Health Serv Res. 2020 Mar 12;20:203. doi: 10.1186/s12913-020-5064-3 (PMC7069220; doi:10.1186/s12913-020-5064-3)
Supplement: Supplementary file 2 — Additional file 2. Intention to engage in IP-SDM among healthcare professionals by discipline at study entry and exit. This table presents median scores of intention by healthcare professionals’ discipline at study entry and study exit. [file 12913_2020_5064_MOESM2_ESM.docx]

**Table:** Intention to engage in IP-SDM among healthcare professionals by discipline at study entry and exit

|  | **Nurses** | **Rehabilitation team** | **Technologists & technicians** | **Social workers** | **Social, community & education paraprofessionals** | **Activities coordinators** |
| --- | --- | --- | --- | --- | --- | --- |
| Intention at entry  (n = 267) | 60;  6 (4.5 – 6.5)* | 50;  6 (5 – 7) | 10;  4.7 (3.5 – 6) | 99;  6 (5 – 7) | 46;  6 (5 – 6.5) | 2;  5.2 (4 – 6.5) |
| Intention at exit  (n=170) | 29;  6 (5 – 7) | 34;  5 (4 – 6.5) | 7;  3.5 (3 – 4) | 75;  5.5 (5 – 6.5) | 23;  6 (5 – 7) | 2;  3.5 (2 – 5) |

*Sample size; median (interquartile range)
